# Growth of high-quality Bi_2_Sr_2_ CaCu_2_O_8+*δ*_ whiskers and electrical properties of resulting exfoliated flakes

**DOI:** 10.1038/s41598-017-03408-2

**Published:** 2017-06-12

**Authors:** Apoorv Jindal, Digambar A. Jangade, Nikhil Kumar, Jaykumar Vaidya, Ipsita Das, Rudheer Bapat, Jayesh Parmar, Bhagyashree A. Chalke, Arumugam Thamizhavel, Mandar M. Deshmukh

**Affiliations:** Department of Condensed Matter Physics and Materials Science, Tata Institute of Fundamental Research, Homi Bhabha Road, Mumbai, 400005 India

## Abstract

In this work, we demonstrate a simple technique to grow high-quality whiskers of Bi_2_ Sr_2_ CaCu_2_ O_8+*δ*_ – a high T_*c*_ superconductor. Structural analysis shows the single-crystalline nature of the grown whiskers. To probe electrical properties, we exfoliate these whiskers into thin flakes (~50 nm thick) using the scotch-tape technique and develop a process to realize good electrical contacts. We observe a superconducting critical temperature, T_*c*_, of 86 K. We map the evolution of the critical current as a function of temperature. With 2-D materials emerging as an exciting platform to study low-dimensional physics, our work paves the way for future studies on two-dimensional high-T_*c*_ superconductivity.

## Introduction

The isolation of graphene and other layered materials like transition metal dichalcogenides has ushered in a new era in the field of two-dimensional physics^1–3^. Mechanical exfoliation of layered materials bonded by van der Waals forces is able to deliver single-crystalline ultraclean material in the monolayer limit. 2D materials are an exciting prospect to study two-dimensional superconductivity. Thin flakes of NbSe_2_, TaS_2_, and FeSe have been discovered to be superconducting, fuelling the progress in this field^4–6^. Efforts have been made to electrostatically induce superconductivity in 2D semiconductors, as well^7, 8^.

Bi_2_Sr_2_CaCu_2_O_8+*δ*_ (BSCCO) has been known as a high-T_*c*_ superconductor and several of its forms including single-crystals, whiskers, and thin films have been grown using different methods including epitaxy, sputtering, and pulsed laser deposition^9–14^. Owing to the layered nature of BSCCO, its exfoliation, akin to graphene, has been recently reported^3, 15–17^. Exfoliation allows us to study variety of physics with choice of substrate by making heterostructures^18^. These techniques have been recently employed to study quantum Hall effect with supercurrent in graphene, and specular interband Andreev reflection in graphene-NbSe_2_ interfaces^19, 20^.

In our work, we present a simple and reproducible method to grow BSCCO whiskers from commercially available powder. Growth of whiskers is preferred due to their single-crystalline nature^10, 21^. As BSCCO is a layered material, we exfoliate its whiskers into thin flakes and perform electrical transport measurements. In previous experiments on exfoliated BSCCO flakes, some groups observed superconductivity^15, 16^, while some did not^3^. This is because of ambient exfoliation and processing that causes the degradation of the top layers of BSCCO thin flakes. To overcome this problem we have developed a fabrication scheme, where the BSCCO flakes are exfoliated inside a glove box in nitrogen environment and the top few layers are removed by *in-situ* Ar etching prior to the metal deposition for electrodes. Here, we observe a superconducting critical temperature, T_*c*_, of 86 K and further observe features of phase-slips in our current-voltage characteristics. The observation of phase-slips makes the system exciting as it provides a pathway to further probe low-dimensional high-temperature superconductivity.

Single-crystalline BSCCO whiskers were prepared by annealing amorphous melt-quenched shards at elevated temperatures in an oxygen atmosphere, this follows the work done earlier^11, 21–27^. However, unlike the previously used complex experimental procedures where whiskers were prepared from carbonates and oxides of Bi, Sr, Ca, and Cu mixed in stoichiometric proportions, we started with commercial BSCCO powder (Sigma Aldrich - 365106). In the review article by P. Bedica *et al*.^21^, various growth methods for the Bi-2212 growth are presented. Here we have compared different works by various groups on melt quenched process in Table 1. Next we discuss the growth procedure which we were able to optimize to develop a repeatable technique. Needless to say we benefit immensely from the past work (as shown in Table 1) of number of researchers before us who used melt quenching to grow high quality whiskers.

**Table 1:** Comparison of Bi-2212 growth by various groups on melt quenched process with the present work.

Article	Growth Criteria	Key of the Growth Mechanism	Outcome
Jung *et al*.^22, 26^	Powder pellets sintering	Inner compressive stress	Bi-2212 growth with *T*_*c*_ = 75 K
Zhou *et al*.^27^	Amorphous glassy pellets sintering	Inner compressive stress	Bi-2212 growth mainly near the surface of the metal quenched
Matsubara *et al*.^23^	Amorphous glassy pellets sintering	Inner compressive stress	Bi-2212 growth with *T*_*c*_ = 70 K and *J*_*c*_ ~ 10^4^–10 A/cm^2^
Ramanathan *et al*.^11^	Amorphous glassy pellets sintering	Inner compressive stress	Bi-2212 growth with needle like grains
Mastubara *et al*.^28^	Annealing Bi- rich melt	Seeded growth	Bi-2212 whiskers grown perpendicular to the glassy substrate
Kraf *et al*.^25^	Melt annealing	Self flux growth	Ribbon like Bi-2212 whiskers with *T*_*c*_ = 80 K
Present work	Commercial BSCCO powder sintering	Inner compressive stress	Single Crystalline Bi-2212, ribbon like whiskers with *T*_*c*_ = 86 K for the exfoliated thin flakes.

**Figure 1:**
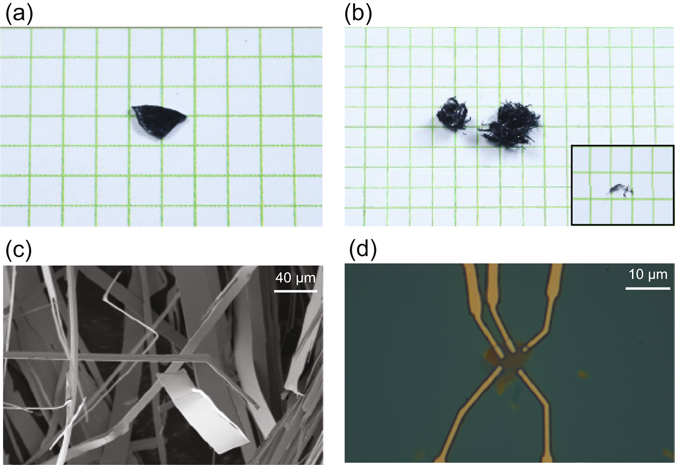
Growth of BSCCO whiskers. (**a**) Photograph of an amorphous BSCCO shard. (**b**) Photograph of grown whiskers on the shard following the annealing. (Inset) Single whisker. Scale - 1 sq. grid = 1 cm^2^. (**c**) SEM image of grown whiskers. (**d**) Optical image of device fabricated to measure electronic properties of exfoliated BSCCO thin flakes (D1).

Firstly, we prepared a BSCCO shard. About 20 g of the BSCCO powder was taken in a high-quality recrystallized alumina crucible and heated to 1100 °C in a resistive-heating box-type furnace. The typical temperature profile for this growth cycle is shown in the Supplementary material. The sample was held at 1100 °C for about 24 hours in order to achieve proper homogenization and then splat quenched between two heavy copper plates, followed by cooling with liquid nitrogen. Subsequently, this BSCCO shard was annealed at 875 °C for 10 days in a 50 sccm oxygen flow (purity of oxygen ~99.5%); the temperature variation in this critical growth cycle is shown in Fig. 1(b) in Supplementary material. Photograph of a typical plate-like shard prior to annealing is shown in Fig. 1(a). Following the annealing process several needle like whiskers were found to grow out of the glass shard as shown in Fig. 1(b). Surface profile of the whiskers is imaged using scanning electron microscopy (SEM). The surface is seen to be flat, in Fig. 1(c), devoid of any visible surface roughness. Figure 1(d) shows the resulting multi-terminal device (D1) that was fabricated to probe the electrical properties of the exfoliated thin flakes; detailed fabrication process is discussed later in this letter.

**Figure 2:**
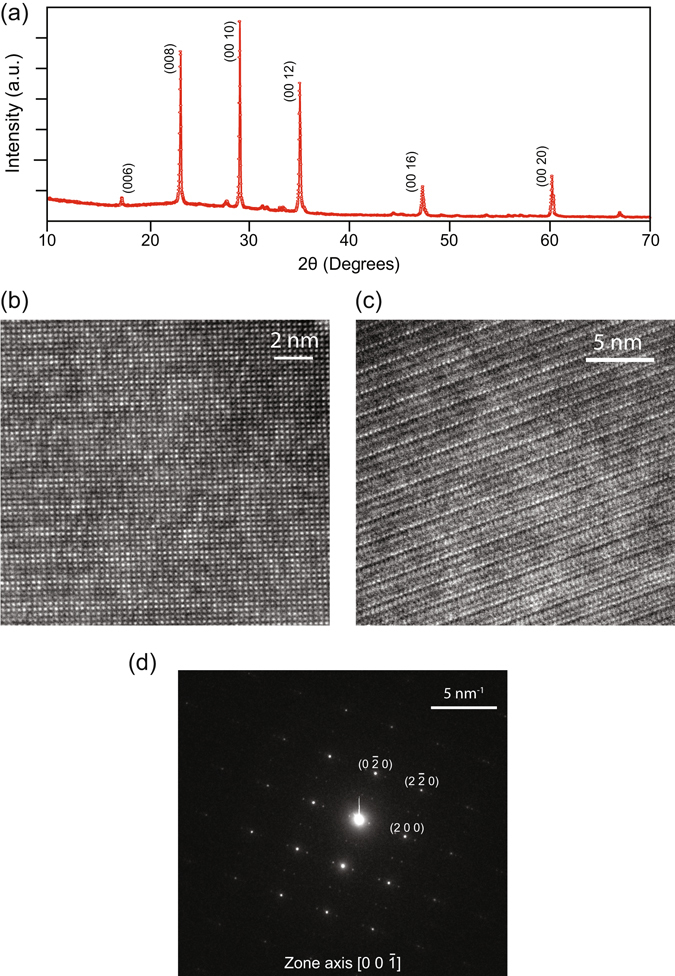
Structural characterization of BSCCO whiskers. (**a**) Powder x-ray diffraction pattern of BSCCO whiskers. The powder diffraction pattern shows (00*l*) plane as the preferred orientation, where the thickness of the whisker corresponds to *c*-plane. (**b**) HRTEM of BSCCO whiskers with incident beam parallel to *c*-axis. (**c**) Cross-sectional TEM with incident beam perpendicular to *c*-axis. Periodic lines at interval of 1.52 nm are visible. (**d**) Electron-beam diffraction with incident beam parallel to *c*-axis. HRTEM and electron-beam diffraction data suggest high crystalline quality of the whiskers.

To check the phase purity of grown whiskers, we isolated a few whiskers, ground them to fine powder, and performed x-ray diffraction (XRD) using a PANalytical x-ray diffractometer. A monochromatic Cu-K_*α*_ (*λ* = 1.5406 Å) wavelength x-ray was used. The obtained XRD pattern of the whiskers is shown in Fig. 2(a). The pattern is well indexed to the crystal structure of BSCCO with the lattice constants *a* = 5.414 Å, *b* = 5.418 Å and *c* = 30.89 Å, and the space group *Fmmm*. Since it is not possible to grind the whiskers to represent all crystal faces, we observe the preferred orientation of grains along the (00l) plane which have a very large intensity compared to others. This also proves that the flat plane of the whiskers, or their thickness, corresponds to the (001) plane. Our lattice constants and XRD pattern are in good agreement with previous results^21, 29^.

The crystal structure of grown BSCCO whiskers was determined using an FEI TECNAI 200 kV Transmission Electron Microscope (TEM). The TEM sample was prepared by gently rubbing an ultrathin carbon grid on the as grown whiskers. Figure 2(b) shows the high-resolution TEM (HRTEM) image of the sample, while Fig. 2(c) presents the cross-sectional TEM of a whisker. We clearly observe individual layers separated by 1.52 nm in the image. Figure 2(d) shows the diffraction pattern taken along the [0 0–1] zone axis. Diffraction pattern was indexed using a JEMS simulation software. Our TEM diffraction analysis corroborates the results of XRD study with an *Fmmm* space group and lattice parameters, *a* = 5.414 Å, *b* = 5.418 Å and *c* = 30.89 Å. From HRTEM images and diffraction pattern, it can be concluded that there are very few defects in the crystal. We also observe satellite spots in the diffraction pattern. These can be attributed to incommensurate modulation in BSCCO structure which have been studied previously^30^. This modulation is also seen in the HRTEM image, Fig. 2(b), and is attributed to bismuth-rich and deficient bands along the *b*-axis^31^.

Energy dispersive x-ray spectroscopy (EDX) was also used to analyze the elemental composition of grown whiskers. An approximate 2:2:1:2:8 ratio of Bi, Sr, Ca, Cu and O, respectively, with ~10% uncertainty was observed. The compositional spectrum values are provided in the Supplementary material.

To probe the electrical properties of the grown whiskers transport measurements on BSCCO thin flakes were done. BSCCO is a layered material like graphene and can be easily exfoliated using the scotch-tape technique; crystals are cleaved along its BiO planes which are bonded by van der Waals forces^32^. BSCCO was exfoliated inside a glove box in nitrogen environment on a p^++^ Si substrate with a 300 nm SiO_2_ capping layer. The detailed exfoliation procedure is discussed in the Supplementary information together with photographs. Thin flakes were identified using optical contrast and atomic force microscope (AFM). The exfoliated BSCCO flakes are quickly coated with the bi-layer resist (EL9/PMMA 950A2) and using standard e-beam lithography we wrote the pattern for multi terminal contact electrodes aligned with the flakes using manual marks. After development we etched the top few layers of the BSCCO thin flake using Ar plasma at 0.025 mbar pressure at 25 Watt for 2–4 minutes in a sputtering chamber and sputtered Ag(45 nm)/Au(50 nm) for electrical contacts. The deposition of electrodes was done without breaking vacuum after the *in-situ* etching in a home built etcher inside the sputtering system. The final pattern as shown in Fig. 1(d), is obtained with lift-off in acetone. To prevent degradation with the ambient, we have coated the fabricated device with a polymer resist, or fully covered it with a thin insulating boron nitride flake using the visco-elastic stamping^33^ with the flake transfer set up kept inside the glove box. Thus the life time of such devices can be enhanced with proper protection from ambient. The contacts thus made are non-ohmic with a two probe resistance of 2–10 k Ω. We found ohmic contacts as well, when we etched 5–6 nm of the flake using Ar plasma. The aspect of the degradation of BSCCO layers is well known^34^ and the process to remove the top insulating layers allows us to fabricate good electrical contacts (see supporting information showing TEM images of amorphous layers at the surface of our flakes). The etch process we have developed prior to deposition of metal is key to achieving good contacts to BSCCO flakes. We now discuss the electrical measurements for three different devices namely D1, D2 and D3 to probe the superconducting properties next.

**Figure 3:**
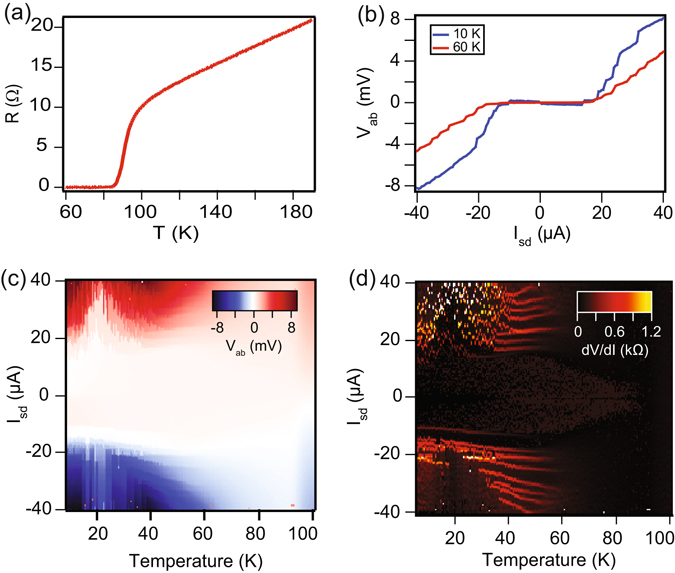
Temperature dependent transport properties, at 0 T, of exfoliated BSCCO flakes. (**a**) Four-probe R vs T plot for the device D2. (**b**) I–V characteristics at 10 K and 60 K for the device D1. A critical current of 13 *μ*A is observed. (**c**) I–V plotted for D1 as a color scale with temperature sweep from 10 K to 100 K. (**d**) Color scale plot of numerical derivative dV/dI from 10 K to 100 K for D1. A superconducting transition temperature (T_*c*_) of 86 K is observed.

Transport measurements in four probe configuration were performed in an Oxford Instruments He^4^ cryostat. The insert was pumped to 10^−6^ mbar prior to measurements. Apart from the temperature sensor in the cryostat, we mounted a calibrated Lakeshore Cernox (CX-1050-AA) on the chip carrier along with the device under test. Figure 3(a) shows the four probe resistance as a function of temperature for the device D2 taken at a bias current of 10 *μ*A. The resistance at room temperature is 30 Ω, which decreases linearly with temperature till 90 K, where the resistance is close to 6 Ω. Thus the residual resistivity ratio (R_300*K*_/R_90*K*_) of the thin flake is close to 5, showing the good quality of the thin film. The onset of superconductivity appears close to 90 K, where resistance drops rapidly and goes to zero at the critical temperature *T*_*c*_ of 86 K, which is consistent with the reported values for Bi-2212 phase of grown BSCCO whiskers^21^; the width of the transition is ~4 K. In addition, the presence of a single *T*_*c*_ indicates the single phase nature of the BSCCO whiskers. Apart from the XRD data, our cross-sectional TEM (see Fig. 2(c)) shows interlayer separation of 1.52 nm which corresponds to that of a half unit-cell for the 2212 phase. We have conducted temperature-dependent electrical measurements on multiple devices and all were found to possess the similar T_*c*_. Figure 3(b) shows the current-voltage characteristics (IVCs) of a BSCCO device, D1, at two different temperatures 10 K and 60 K. The observed critical current, I_*c*_, at 10 K is 13 *μ* A, which corresponds to the critical current density (J_*c*_) of 3095 A/cm. Figure 3(c) presents the IVCs measured for a temperature range from 10 K to 100 K. To find the superconducting transition temperature (*T*_*c*_), we plot the numerical derivative (dV/dI vs I) for the entire temperature range (10 K to 100 K) as shown in Fig. 3(d); here V is the four probe voltage measured across the inner two probes and I is the current through the device. Below 86 K, we observe a finite supercurrent confirming our T_*c*_ to be close to 86 K which is consistent with the R vs T measurement as shown in Fig. 3(a). Next we discuss the I-V response as the system is driven from a superconducting to normal state with a focus on – a) the magnitude of the critical current density, and b) the origin of the hysteretic response.

**Figure 4:**
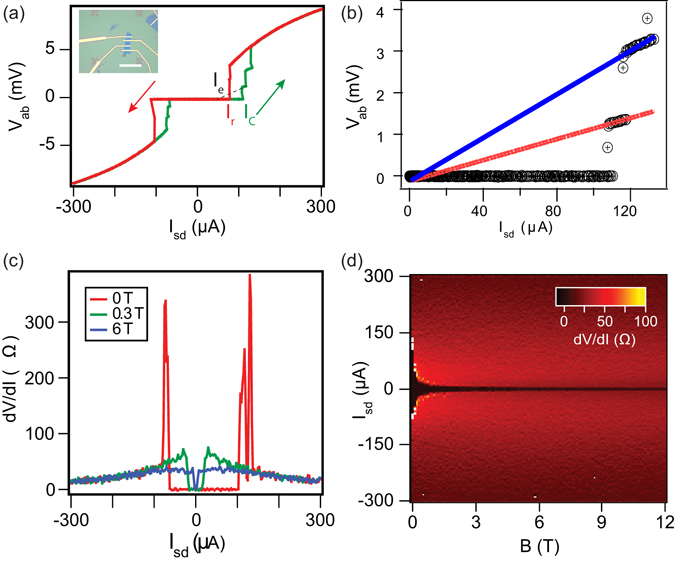
Magnetic field dependent transport properties of exfoliated BSCCO flakes for the device D3. (**a**) Zero field four-probe I-V characteristics at 10 K. (Inset) Optical Image for Device D3. Scale bar corresponds to 40 *μ*m. (**b**) Similar excess current for the two voltage jumps in IVC taken at 10 K. (**c**) Numerical derivative of IVCs at various B. (**d**) Colorscale plot of numerical derivative, dV/dI as a function of I, from 0 T to 12 T. Superconductivity is observed even at 12 T magnetic field. All magnetic field measurements were performed at 10 K.

Figure 4(a) shows the IVC at 10 K at zero-field for D3 (device image in inset). We find from data shown in Figs 3(b) and 4(a) that J_*c*_ is significantly lower (3.1 × 10^3^ A/cm^2^ for D1 and 2.7 × 10^4^ A/cm^2^ for D3) than those reported for bulk whiskers and epitaxial thin films^21, 35^ which are in the range of 10^5^–10^6^ A/cm^2^. For D2 we have observed a *J*_*c*_ value of 5.0 × 10^5^ A/cm^2^ at 10 K. Thus we do see a variation in *J*_*c*_. The *J*_*c*_ values in the range of 10^2^–105 A/cm^2^ have earlier been observed for Bi-2212 thin films^15, 23, 36, 37^. Possibly, there are several mechanisms at play that could result in a lower J_*c*_ – firstly, insulating regions near surface are formed due to exposure to ambient^34, 38^, thereby reducing the effective conducting thickness. Secondly, for high - T_*c*_ superconductors, rising defect density has been shown to contribute to a higher J_*c*_^39^^–^^41^. Our samples are seen to be nearly defect-free in TEM images. This could also be a reason for a lower J_*c*_. Balestrino *et al*.^37^ shows that substrate too plays a role in defining *J*_*c*_. So role of SiO_2_/Si substrate too needs further investigation. Lastly, exposure to chemicals during lithography could also play a role in the reduction of J_*c*_. This variation in *J*_*c*_ is also observed in the literature without complete microscopic understanding. Thus at present the microscopic mechanism for variation in *J*_*c*_ is not quite clear to us. Further detailed work needs to be done to elucidate the mechanism for the low critical current density. A lower J_*c*_, however, is very well-suited for integrating BSCCO flakes with other 2D materials to form mesoscopic devices like DC nano-SQUIDs. These whiskers and resulting flakes provide us a comprehensive platform to probe transport and associated physics in such superconductor-2D material interfaces and junctions. In the following part, we discuss the origin of the hysteretic response of the IVCs.

The IVC shown in Fig. 4(a) is hysteretic in nature. While ramping the current up, the device switches from a zero voltage state to a finite voltage state at the critical current I_*c*_ = 110 *μ*A. Apart from the resistive jump at I_*c*_, we see multiple voltage steps in IVC, till the entire device transits to a completely normal state. While ramping the current down, the device switches back to zero resistance state at a current lower than I_*c*_ known as the retrapping current, I_*r*_ = 78 *μ*A. The origins of I_*c*_ and I_*r*_ are different; I_*c*_ is dictated by superconducting coherence, while I_*r*_ is due to Joule heating which is well understood for the case of superconducting microbridges^42^, weak links^43^, and nanowires^44^.

Apart from the resistive jump at I_*c*_, we see multiple steps in IVCs shown in Figs 3(b) and 4(a), which resemble phase-slip features^45–48^. At these phase-slip features, the order parameter fluctuates locally and goes to zero, while the phase of the order parameter slips by 2 *π*, resulting in a finite voltage state. These features are seen to last till 70 K while sweeping up the temperature [see Fig. 3(c) and (d)]. By extrapolating the resistive slope above each voltage jump in IVC shown in Fig. 4(b), we obtain a finite excess current (I_*e*_) – the intercept on the current axis, which is same for the two voltage steps, indicating homogeneity of the BSCCO flake. The voltage steps in IVCs originated due to phase slips, result in sharp peaks in its numerical derivative plot as observed in Fig. 4(c).

Voltage steps in IVCs and the appearance of similar excess current for each phase slip have been modelled by Skocpol *et al*.^49^ for superconducting tin micro-bridges. In this model the behavior of phase slip centers (PSCs) has been discussed, which appear in a localized region of coherence length, *ξ*, although the measured voltage drop is dependent on the much larger quasi-particle diffusion length scale (Λ). The height of the voltage step is given as V_*step*_ = 2 Λ R_*N*_(I − I_*e*_)/ L; where, R_*N*_ is the normal-state resistance, I is the value of current at the phase-slip, and L is the distance between voltage probes. The estimated Λ for our flakes from the IVC fit is 2.2 *μ*m. The width of our device is larger than the estimated Λ, so we are not strictly in the one dimensional limit. Experiments on superconducting thin films present a model for phase slips in 2-D superconductors analogous to PSCs in 1-D superconductors^46, 47^. It is argued that the phase slips demonstrate a similar behaviour as PSCs, but the central core is replaced by a line crossing the film width and is known as a phase slip line (PSL). The estimated value of Λ indicates that lithographic techniques can be used to pattern BSCCO flakes to reach the one dimensional limit.

We also performed transport measurements in presence of a perpendicular magnetic field while maintaining a bath temperature at 10 K on D3 (magnetic field dependence on the transport of D1 is shown in Supplementary material). To study the evolution of the critical current as a function of the magnetic field, *B*, we plot the numerical derivative of the IVCs (Fig. 4(c) and (d)). An I_*c*_ of 102 *μ*A is observed in zero field which reduces to 15 *μ*A at 0.3 T, and 2.5 *μ*A at 6 T as shown in Fig. 4(c); (additional information about the variation of critical current as a function of field is presented in the supporting information). A finite supercurrent is observed at a 12 T magnetic field, as well. We infer a high critical field for the BSCCO flakes, as would be expected from high quality crystals.

In summary, we have presented a simple growth method to obtain high-quality single-crystalline BSCCO whiskers. Using our method, one can grow BSCCO whiskers directly from commercially available powder, and we do not need to start with individual oxides or carbonates of Bi, Sr, Cu, and Ca mixed in stoichiometric proportions. Whiskers grown are seen to be upto 1 cm long and have a clean surface. They are also seen to have low defect density and correspond to the 2212 phase of BSCCO. For electrical characterization, we exfoliate the whiskers into thin flakes and observe a T_*c*_ close to 86 K. To fabricate the devices we develop a process to make electrical contacts by *in-situ* etching prior to metal deposition. We also observe signatures of phase-slips in our current-voltage characteristics. The low critical current density for our BSSCO whiskers indicates that it may be possible to fabricate mesoscopic devices like DC SQUIDs for sensing applications at high temperatures and high magnetic fields.

## Electronic supplementary material

Supplementary Material
