# Supplementary material for: Growth of high-quality Bi2Sr2 CaCu2O8+δ whiskers and electrical properties of resulting exfoliated flakes
Source: Sci Rep. 2017 Jun 12;7:3295. doi: 10.1038/s41598-017-03408-2 (PMC5468279; doi:10.1038/s41598-017-03408-2)
Supplement: Supplementary file 1 — Supplementary Material [file 41598_2017_3408_MOESM1_ESM.pdf]

Growth of high-quality $Bi_2Sr_2CaCu_2O_{8+\delta}$ whiskers and electrical properties of resulting exfoliated flakes

Apoorv Jindal,[1,*] Digambar A. Jangade,[1,*] Nikhil Kumar,[1,*] Jaykumar Vaidya,[1] Ipsita Das,[1] Rudheer Bapat,[1] Jayesh Parmar,[1] Bhagyashree A. Chalke,[1] Arumugam Thamizhavel,[1,†] and Mandar M. Deshmukh[1,‡]

[1]*Department of Condensed Matter Physics and Materials Science, Tata Institute of Fundamental Research, Homi Bhabha Road, Mumbai - 400005, India*

(Dated: April 14, 2017)

* These authors contributed equally

† thamizh@tifr.res.in

‡ deshmukh@tifr.res.in

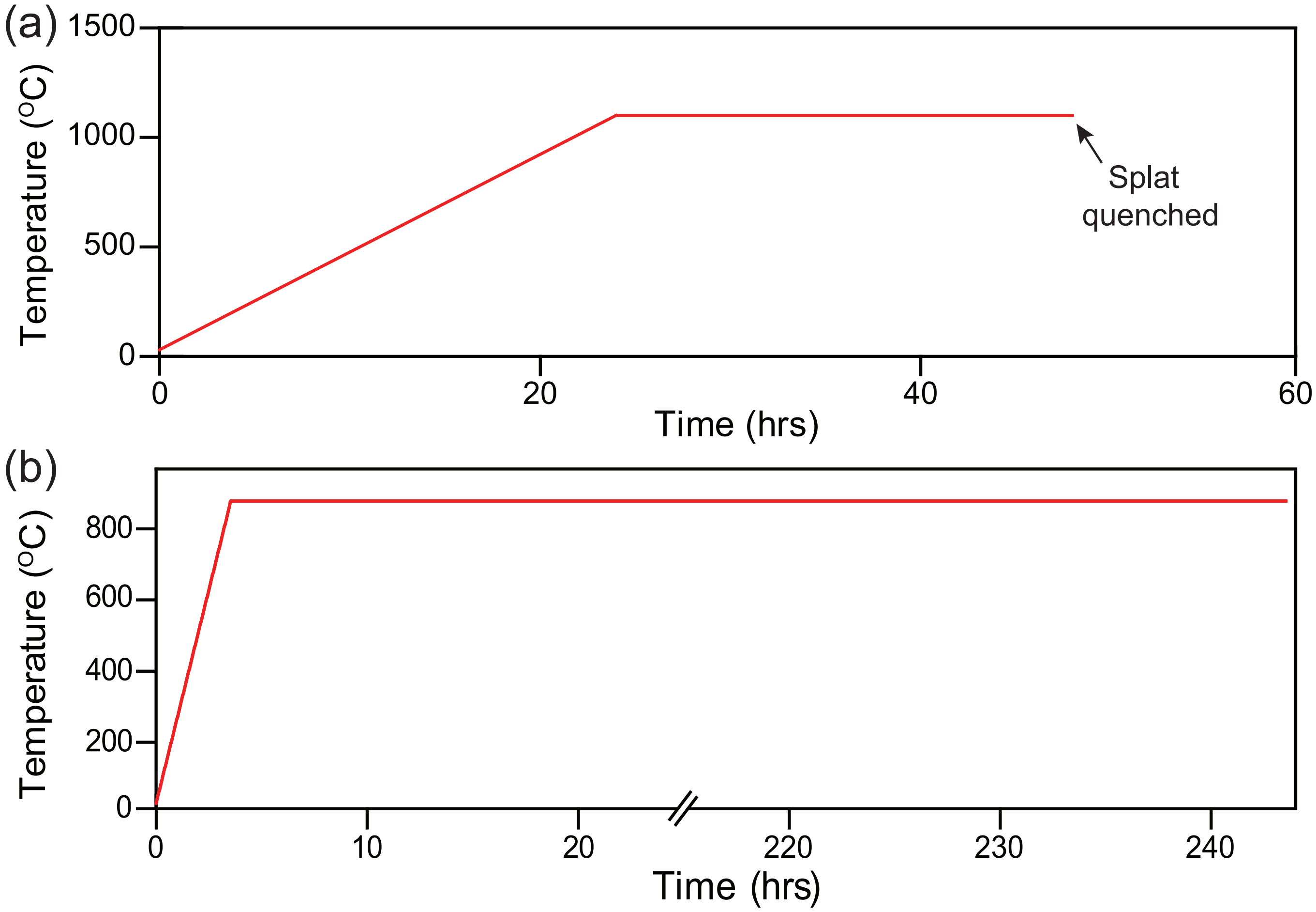

FIG. 1. Growth of BSCCO whiskers. (a) Temperature profile for synthesizing an amorphous BSCCO shard, (b) Temperature evolution during the annealing of the shard in a tube furnace to grow whiskers. Annealing was performed under a constant flow of 50 sccm oxygen.

I. GROWTH TEMPERATURE VARIATION

For the BSCCO shard preparation, the BSCCO powder is first heated to 1100 °C in an alumina crucible in a resistive-heating box-type furnace. The temperature profile is shown in Figure 1(a). Here the sample was held at 1100 °C for about 24 hours in order to achieve proper homogenization and then splat quenched as discussed in the main text. Figure 1(b) shows the temperature profile, where BSCCO shard was annealed at 875 °C for 10 days in a 50 sccm oxygen flow.

II. EXFOLIATION PROCESS

We have exfoliated the thin flakes of BSCCO from the grown whiskers using scotch tape inside the glove box filled with nitrogen atmosphere. We have followed the standard recipe that is being used to exfoliate Graphene and other 2D materials (see reference 3, 15 and 17 of the main paper) and have shown various steps in Fig 2 of this supplementary information. In this recipe, we put few grown whiskers on the adhesive scotch tape and peeled off many times. Finally the thin flakes of BSCCO are transferred on the oxygen plasma cleaned SiO_2/Si substrate by gently rubbing the tape with the backside of a dropper.

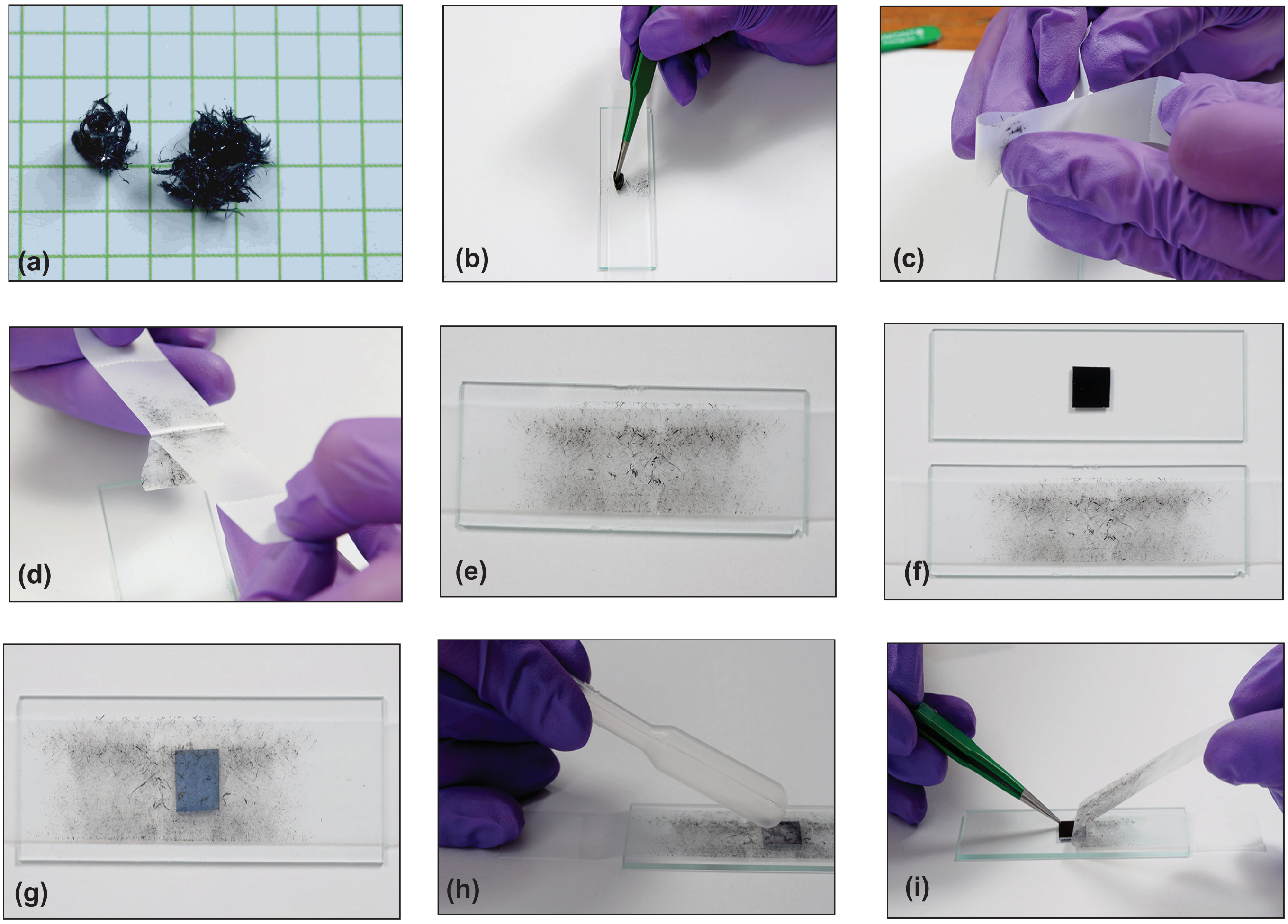

FIG. 2. Exfoliation steps for BSCCO whiskers. (a) The grown whiskers (b) The grown whiskers adhere to the scotch tape, kept on top of a glass slide. (c)-(e) The peeling off the scotch tape to get uniform crystal density in the mid of the tape. (f) The final tape with uniform crystal density and the SiO_2/Si substrate kept on another glass slide.(g) The scotch tape is now kept on top of an oxygen plasma cleaned SiO_2/Si substrate. (h) Gentle rubbing the tape through a dropper so that the thin flakes are transferred on the substrate. (i) Peeling off the scotch tape from the substrate with the help of a tweezer which leaves exfoliated flakes on the substrate.

III. DEVICE DIMENSIONS

In this paper, we have presented the characteristics of the three different BSCCO thin flake devices, namely D1, D2 and D3. The dimensions of these devices are mentioned below in table I.

Device	Length (μm)	Width (μm)	Thickness (nm)	Critical Current Density (A/cm^2)
D1	5.2	6.3	50	3.0×10^3
D2	17.2	28.4	50	5.0×10^5
D3	19.6	8.6	47	2.7×10^4

TABLE I. Comparison of various devices, presented in this work.

IV. EDX COMPOSITION SPECTRUM

Figure 3 shows the compositional spectrum captured by energy dispersive X-ray spectroscopy (EDX) analysis of the grown whiskers. Compositional values normalized to Oxygen are presented in table II, which shows an approximate

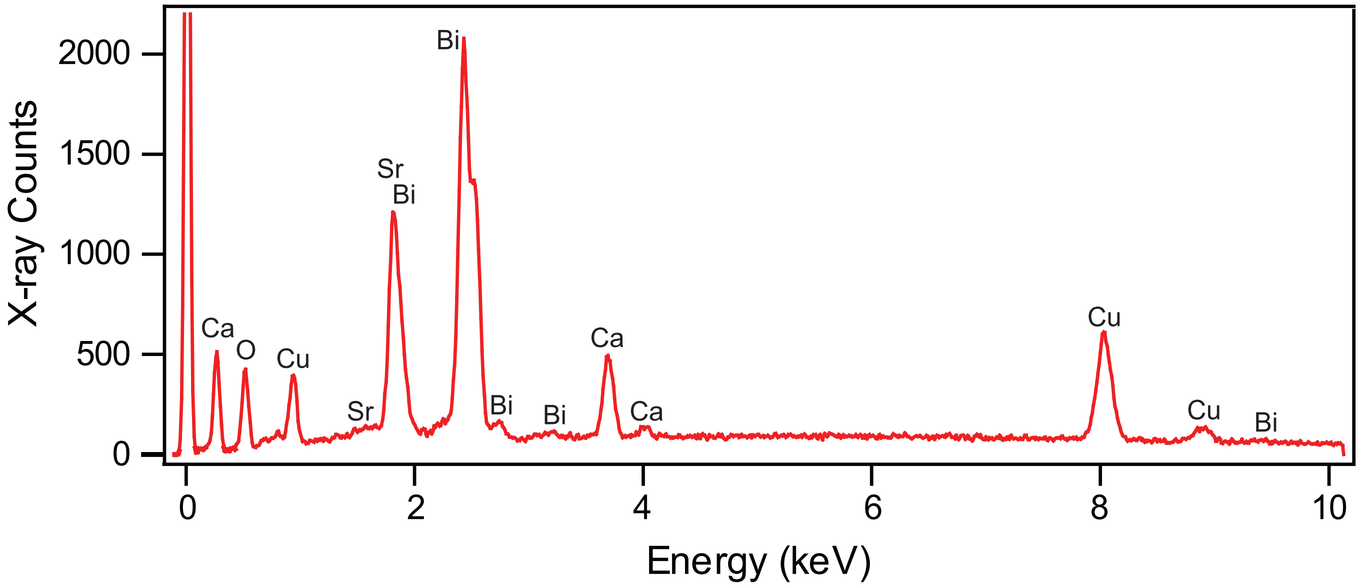

FIG. 3. Energy dispersive x-ray spectroscopy (EDX) spectrum for our whiskers confirming the 2212 stoichiometry.

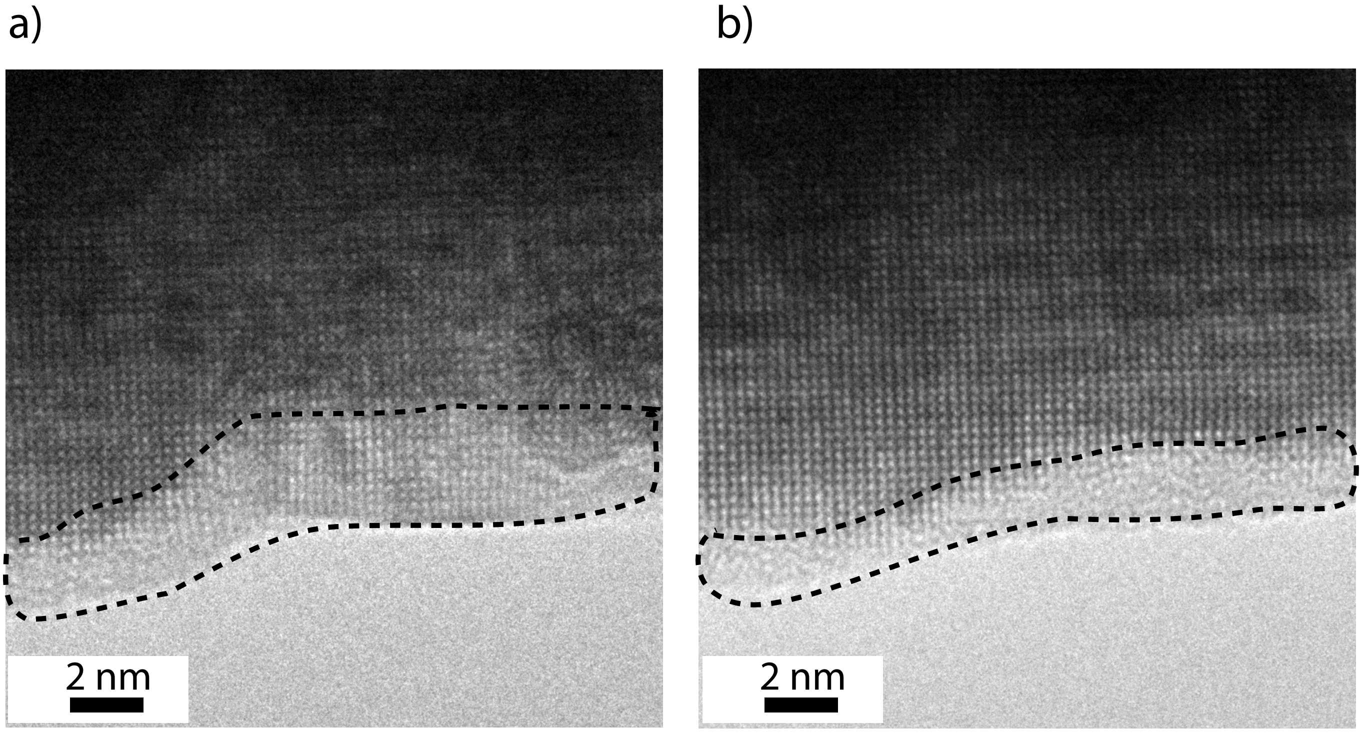

FIG. 4. HRTEM images of the grown whiskers showing amorphous layers at the surface of our flakes taken at two different positions.

2:2:1:2:8 ratio of Bi, Sr, Ca, Cu and O, respectively, with ∼10% uncertainty. This confirms the 2212 stoichiometry for our grown whiskers.

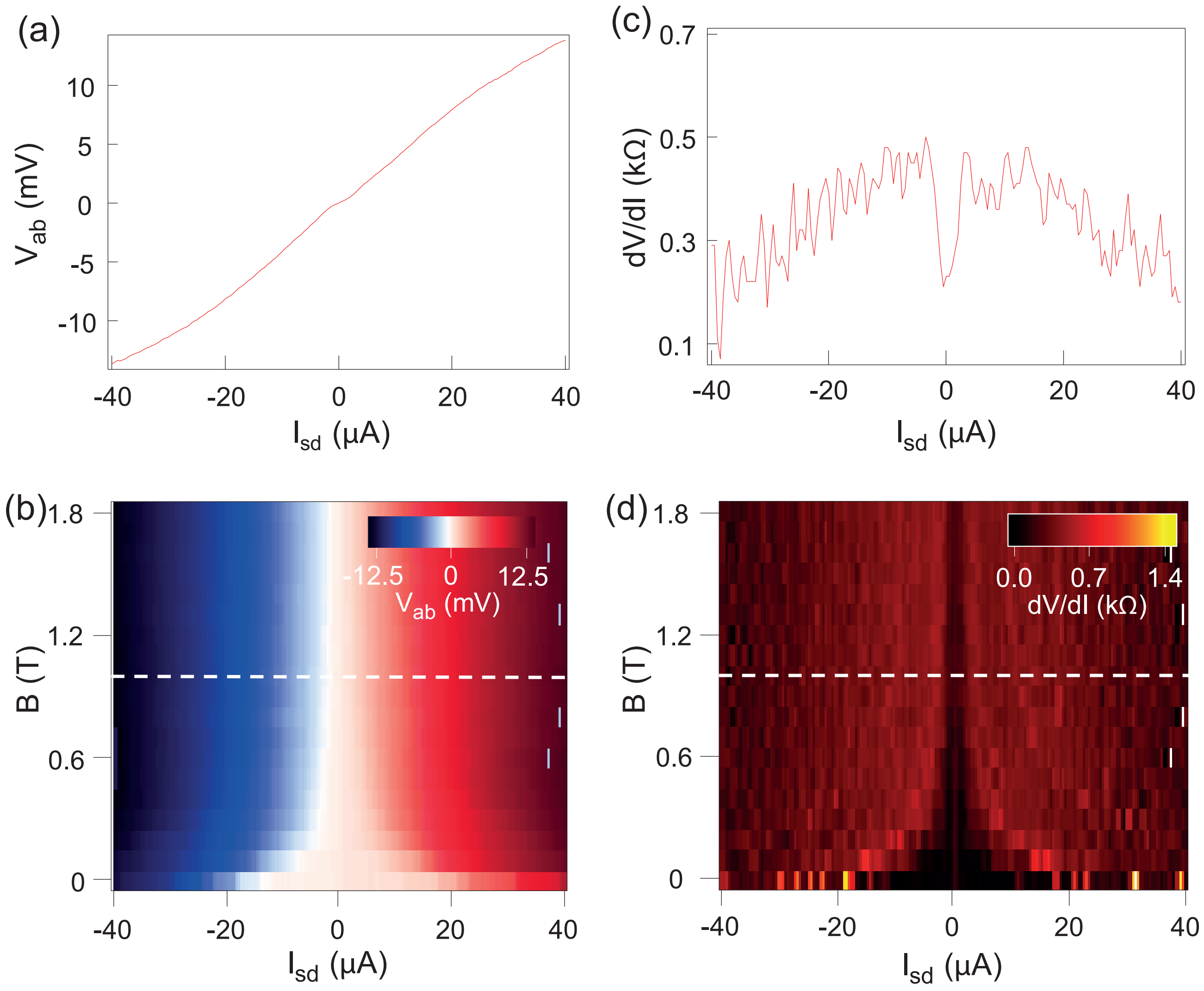

FIG. 5. Magnetic field dependent properties for D1. (a) Four-probe V-I characteristic at B = 1 T. (b) Magnetic field sweep from 0 T to 2 T. (c) Numerical derivative of IVCs at B = 1 T. (d) Numerical derivative of IVCs, dV/dI, from 0 T to 2 T. All magnetic field measurements were performed at 10 K.

Spectrum	O	Ca	Cu	Sr	Bi
Spectrum 1	51.44	6.10	18.12	10.54	13.80
Spectrum 2	62.97	5.45	9.79	9.56	12.24

TABLE II. Compositional values normalized to oxygen

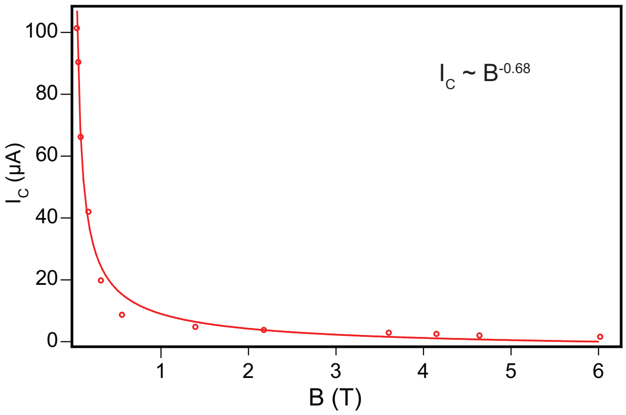

FIG. 6. Fitting for I_c with B at 10 K. A power-law relation is observed with $I_c \propto B^{-0.68}$.

V. TEM IMAGE SHOWING AMORPHOUS LAYERS AT THE TOP SURFACE

Figure 4 presents the HRTEM images of the grown whisker showing the top surface of the flake. The dotted region shows the amorphous layers at the top surface of the flake. This gives a sense of the degraded region with exposure to ambient.

VI. MAGNETIC FIELD MEASUREMENTS FOR D1

Figure 5 shows the magnetic field dependent IVCs for D1 taken at 10K. The IVC at 1T field is presented in Figure 5(a), showing a non zero critical current. We swept the magnetic field from 0T to 2 T, and captured the IVCs at a fixed temperature of 10K [see Figure 5(b)]. The critical current at zero filed is 40 μA and it decreases rapidly with the applied magnetic field as shown in Figure 5(b). The numerical derivative (dV/dI) of IVC at 10 K temperature and 1T magnetic filed is plotted in Figure 5(c). The spike like features at various currents are originated due to the phase slips. Figure 5(d) shows the numerical derivative (dV/dI) of IVC at 10 K temperature at varying magnetic field from 0 to 2 T. From Figure 5(b) and (d), we do see a non-zero critical current even at 2T, showing the critical magnetic field to be larger than 2T.

VII. MAGNETIC FIELD MEASUREMENTS FOR D3

From the magnetic field measurements on D3, discussed in the main text, we observe the critical current (I_c) to decay with increasing magnetic field (see Figure 6). Fitting the I_c with the magnetic field, we get a power-law relation between the two with $I_c \propto B^{-0.68}$. This kind of behaviour has been previously observed in BSCCO, qualitatively, by Schmitt *et al.* for low magnetic fields ($B < 2$ T) [1]. Our I_c - B observations also match with predictions for high-T_c superconductors, especially BSCCO at $T < 20$ K, where the I_c drops quickly for low fields while remaining virtually unchanged for a large magnetic field before going to zero at the critical field [2]. Our measurements are consistent with the high critical magnetic field observed for bulk BSCCO crystals.

---

[1] Schmitt, P., Kummeth, P., Schultz,L., and Saemann-Ischenko, G. Two-dimensional behavior and critical-current anisotropy in epitaxial $Bi_2Sr_2CaCu_2O_{8+x}$ thin films, *Phys. Rev. Lett.* **67,** 267 (1991).
[2] Dew-Hughes, D. The critical current of superconductors: an historical review, *Low Temperature Physics* **27,** 713 (2001).
